# Serum HBV RNA correlated with intrahepatic cccDNA more strongly than other HBV markers during peg-interferon treatment

**DOI:** 10.1186/s12985-020-01471-2

**Published:** 2021-01-06

**Authors:** Xiaomei Wang, Xiumei Chi, Ruihong Wu, Hongqin Xu, Xiuzhu Gao, Lei Yu, Longgen Liu, Mingxiang Zhang, Youwen Tan, Junqi Niu, Qinglong Jin

**Affiliations:** 1 Department of Hepatology, The First Hospital of Jilin University, 71 Xinmin Street, Changchun, 130021 Jilin Province China; 2 Department of Infectious Diseases, The Fourth Hospital of Harbin Medical University, Harbin, 150001 Heilongjiang Province China; 3 Department of Infectious Diseases, The Third Hospital of Changzhou, Changzhou, 213001 Jiangsu Province China; 4 Department of Hepatology, Shenyang Sixth People’s Hospital, Shenyang, 110006 Liaoning Province China; 5 Department of Hepatology, The Third People’s Hospital of Zhenjiang, Zhenjiang, 212021 Jiangsu Province China

**Keywords:** Hepatitis B virus, HBV RNA, HBcrAg, HBV DNA, cccDNA, Interferon

## Abstract

**Background:**

Serum hepatitis B virus RNA (HBV RNA) has been reported to be a surrogate marker of intrahepatic cccDNA during nucleos(t)ide analogs therapy. However, in HBeAg-positive patients treated with peg-interferon (peg-IFN), whether HBV RNA is superior to other HBV markers in reflecting cccDNA profile is still unclear.

**Methods:**

Serum HBV RNA, HBcrAg, HBV DNA, and HBsAg were longitudinally assessed among 30 HBeAg-positive patients during 48-week peg-IFN treatment. Besides, intrahepatic cccDNA was detected at baseline and week 48 respectively. Then, the individual correlations between HBV RNA, HBcrAg, HBV DNA, HBsAg, and cccDNA were statistically analyzed.

**Results:**

HBV RNA levels decreased more rapidly in patients with HBeAg seroconversion than those without HBeAg seroconversion. Among all patients, cccDNA correlated better with HBV RNA than with HBcrAg, HBV DNA, and HBsAg at baseline. After 48 weeks peg-IFN treatment, cccDNA still correlated more strongly with HBV RNA than other HBV markers. Further analysis indicated that in patients with HBeAg seroconversion cccDNA strongly correlated with HBV RNA and HBcrAg, whereas not correlate with HBV DNA and HBsAg. While in patients without HBeAg seroconversion, cccDNA highly correlated with HBV RNA and HBV DNA, moderately correlated with HBcrAg, and not correlated with HBsAg.

**Conclusion:**

Compared to HBcrAg, HBV DNA, and HBsAg, serum HBV RNA correlated more strongly with intrahepatic cccDNA levels before and after 48-week peg-IFN treatment. The level of serum HBV RNA may be a superior surrogate marker in reflecting the intrahepatic cccDNA profile in HBeAg-positive patients during peg-IFN treatment.

*Trial registration* ClinicalTrials, NCT03546530. Registered 1 January 2015. https://clinicaltrials.gov/ct2/results?cond=&term=NCT03546530.

## Background

Hepatitis B virus (HBV) infection is a major global health problem. Chronic HBV infection greatly increases the risk of terminal liver diseases, such as cirrhosis, hepatic decompensation, and hepatocellular carcinoma, which contribute to more than 780,000 deaths every year worldwide [1, 2]. Currently, the goal for the treatment of HBV is to suppress viral replication to the lowest possible level thus halting disease progression. Nowadays, the approved agents for the treatment of chronic HBV infections mainly belong to two classes: pegylated interferon (peg-IFN) and nucleotide/nucleoside analogs (NAs) [3]. Among these agents, peg-IFN, which has the effects of suppressing HBV replication and immunomodulation, is still widely used in the clinic as the first-line therapeutic option for patients with HBeAg-positive chronic hepatitis B (CHB) [4].

Although antiviral agents can effectively reduce the serum HBV DNA level in CHB patients, complete elimination of HBV is difficult due to the existence of intrahepatic covalently closed circular DNA (cccDNA) [5]. HBV cccDNA, which serves as the template for the transcription of a 3.5-kb pregenomic RNA, can produce progeny viral DNA and proteins even in the absence of detectable HBV DNA or HBsAg in the blood [6–8]. Low levels of intrahepatic cccDNA predict a sustained virologic response after cessation of antiviral treatment. Therefore, quantitation of intrahepatic cccDNA is suggested to be a valuable marker in evaluating the cure of CHB and assessing treatment endpoints. However, the invasive nature of liver biopsy is the major obstacle for quantitation of cccDNA, which greatly limits the use of cccDNA as a marker in real-world clinical practice. Therefore, finding non-invasive surrogate markers of intrahepatic cccDNA is clinically meaningful.

Several traditional serum markers, such as quantitative HBsAg and HBV DNA have been proposed to reflect the intrahepatic cccDNA profile [9, 10]. However, the correlations between cccDNA and those traditional markers are too weak to apply them in clinical practice [11].

Recently, serum HBV RNA, which is transcribed from cccDNA, has been reported as a potential intrahepatic cccDNA surrogate marker in CHB patients, as well as during NAs therapy [12–14]. Serum HBV RNA was also identified as a good predictor of HBeAg seroconversion in HBeAg-positive patients during therapy with peg-IFN [4]. In addition, a mouse model experiment revealed that HBV RNA correlated positively with cccDNA before treatment, whereas no correlation between them after 6 weeks of peg-IFN therapy [12].

HBV core-related antigen (HBcrAg) is another new potential marker of HBV infection, which consists of three species of related proteins, including hepatitis B core antigen, hepatitis B e antigen, and a truncated 22KDa precore protein [15]. Serum HBcrAg was also considered to be correlated with cccDNA activity and a good biomarker in predicting HBeAg seroconversion in patients treated with peg-IFN [16, 17].

Despite the findings above, it remains unclear whether HBV RNA or HBcrAg could offer better predictive performance compared with traditional serum viral markers such as HBV DNA or HBsAg in reflecting the intrahepatic cccDNA among HBeAg-positive patients during peg-IFN treatment.

### Aim of the study

In the present study, our aim was to find a better surrogate marker of cccDNA in HBeAg-positive patients with peg-IFN treatment through evaluating the correlations of HBV RNA, HBcrAg, HBV DNA, and HBsAg with cccDNA. At the same time, the dynamic changes of serum HBV RNA and HBV DNA were also investigated.

## Materials and methods

### Study participants

A total of 30 HBeAg-positive, noncirrhotic CHB patients were recruited from a multicenter, randomized, controlled clinical trial between Mar. 2015 and Dec. 2017 (ClinicalTrials.gov Identifier: NCT03546530). All recruited patients completed the full course of 48 weeks treatment, also had paired liver biopsy at baseline and week 48 and serial serum during treatment. The patients were treated with peg-IFN (Kawin Technology, China) at a dosage of 1.5 μg/kg body weight once weekly for 48 weeks. Briefly, the inclusion criteria were as follows: 18 to 60 years old; positive HBsAg for at least 6 months; HBeAg-positive and anti-HBe negative; HBV DNA ≥ 10^5^ IU/mL; ALT ≥ 2 and < 10 × the upper limit of normal; no treatment history. The criteria for exclusion were: positivity for antibodies against HCV, HDV, or HIV; other inflammatory diseases such as rheumatoid arthritis, diabetes, or autoimmune hepatitis; hypertension; kidney disease; or a recent history of infectious disease.

The research protocol was approved by the Ethics Committee of the First Hospital of Jilin University and other participating institutions, according to the Helsinki Declaration of 1975. Written informed consent forms were obtained from all patients.

### Samples

Peripheral serum samples obtained from all patients at baseline, week 4, week 12, week 24, week 36, and week 48 during treatment were stored at − 80 °C for virological analyses. Paired liver biopsies were collected at baseline and week 48 and were snap frozen in liquid nitrogen until cccDNA analyses.

### Standard laboratory assessments

Serum HBV DNA was quantified by quantitative polymerase chain reaction (qPCR) using a Roche COBAS AmpliPrep/COBAS TaqMan system (Roche Diagnostics, Mannheim, Germany) with a lower detection limit of 20 IU/mL. HBV genotypes were determined by real-time PCR with a commercial kit (Shanghai ZJ Bio-Tech, China). HBsAg, HBeAg, anti-HBs, anti-HBe, and anti-HBc were quantitated by chemiluminescence microparticle immunoassays using the Architect i2000SR platform and Abbott Architect reagents (Abbott Laboratories, Chicago, IL) according to manufactures’ instruction. Laboratory assessments were performed at baseline, week 4, week 12, week 24, week 36, and week 48.

### Serum HBV RNA quantification

Serum HBV RNA was quantitatively measured in the period of therapy, at baseline, week 4, week 12, week 24, week 36, and week 48 by previously reported methods [18, 19]. Briefly, HBV RNA was extracted from 200 µL of serum, and then incubated at 37 °C for 30 min with DNase I to digest HBV DNA. cDNA was synthesized using a Transcriptor First Strand cDNA Synthesis Kit (Roche, Mannheim, Germany). Then, quantitative PCR was performed with 2 × RealStar Power Probe Mixture (Gen Star, Beijing, China) with a linear range of 5 × 10^3^ copies/mL to 5 × 10^9^ copies/mL and a lower detection limit of 1 × 10^3^ copies/mL.

### Intrahepatic HBV cccDNA quantification

Intrahepatic HBV cccDNA was quantified by fluorescent probe quantitative PCR assays (SUPBIO Biotechnology, Guangzhou, China). DNA was extracted from approximately 5 mg of frozen liver biopsy tissue using a DNeasy Blood & Tissue Kit (QIAGEN, Hilden, Germany) according to the manufacturer's instructions and then the DNA was denatured at 85 °C for 5 min. Plasmid-safe ATP-dependent DNase was used to digest HBV rcDNA, replicative dsDNA, and ssDNA [20]. HBV cccDNA was quantified by qPCR with a primer pair and a probe targeting the gap region of the HBV genome. The quantification range of cccDNA was 10 to 1 × 10^6^copies/uL. For normalization of the number of intrahepatic cccDNA, a set of primers and a probe were used to quantitatively detect the human β-globin gene by qPCR [19].

### Serum HBcrAg quantification

HBcrAg was measured using a fully automated Lumipulse chemiluminescence enzyme immunoassay (CLEIA) analyzer (Fujirebio Inc., Tokyo, Japan), according to the manufacturer’s instructions. Since the general analytic measurement range of this assay is from 1000 U/mL (3log10 U/mL) to 1 × 10^7^U/mL (7log10 U/mL), serial dilutions of the serum samples are required when the serum qHBcrAg level is above the detection limit of the assay.

### Statistical analysis

The characteristics of the study participants are presented as the proportions, means, or medians. Comparisons between the patients with HBeAg seroconversion and without HBeAg seroconversion were performed using the chi-squared test for categorical data and the Mann–Whitney U-test for continuous data. Serum HBV RNA, HBcrAg, HBV DNA, HBsAg, and intrahepatic cccDNA were expressed in the logarithm. Differences in mean log-transformed values were calculated using Student’s t-test or one-way ANOVA where it is appropriate. The Pearson’s correlation analysis was applied to the log-transformed values of intrahepatic and serum markers. *P* values of < 0.05 were considered significant. Analyses were performed using the statistical software SPSS version 18 for Windows PC (SPSS, Chicago, IL, USA).

## Results

### Patient characteristics at baseline

Our study cohort consisted of 30 patients (18 males and 12 females). The mean age was 26.57 ± 4.17 years. After 48 weeks of peg-IFN treatment, 9 of the 30 patients achieved HBeAg seroconversion. For further comparison, the patients were categorized into two groups: SR group (patients achieving HBeAg seroconversion response, n = 9), NSR group (patients not achieving HBeAg seroconversion response, n = 21).

As shown in Table 1, the comparison of the baseline values between the SR and NSR groups are listed as follows: HBV RNA (7.72 ± 1.42 log_10_ copies/mL vs*.* 7.75 ± 1.09 log_10_ copies/mL, *P* = 0.959), cccDNA (median 30.0 copies/cell vs*.* median 34.4 copies/cell, *P* = 0.946), HBcrAg (5.24 ± 1.45 log_10_ KU/mL vs*.* 5.48 ± 1.16 log_10_ KU/mL, *P* = 0.636), HBV DNA (7.52 ± 1.05 log_10_ IU/mL vs*.* 7.59 ± 1.15 log_10_ IU/mL, *P* = 0.878), and HBsAg (3.59 ± 0.49 log_10_ IU/mL vs*.* 3.86 ± 0.69 log_10_ IU/mL, *P* = 0.310). From the above results, it could be seen that there were no significant differences in HBV RNA, cccDNA, HBcrAg, HBV DNA, and HBsAg at baseline between the SR and NSR groups. In addition, no significant differences in age, HBV genotype, or ALT (all *P* > 0.05) were observed between the two groups.

**Table 1:** Baseline characteristics of all peg-IFN-treated CHB patients

Characteristic	All	SR group	NSR group	*P* value
	(N = 30)	(N = 9)	(N = 21)	(SR vs. NSR)
Age, year (median)	26.57 ± 4.17	27.00 ± 3.08	26.38 ± 4.62	0.216
Gender (male/female)	18/12	8/1	10/11	0.03
HBV genotype (%)				0.614
B	10 (33.3%)	3 (33.3%)	7 (33.3%)	
C	20 (66.7%)	6 (66.7%)	14 (66.7%)	
ALT (U/L, median (range))	120 (66.8–174.0)	131.0 (96.7–181.0)	115.0 (59.0–174.0)	0.380
HBV DNA (log_10_ IU/mL)	7.57 ± 1.10	7.52 ± 1.05	7.59 ± 1.15	0.878
HBsAg (log_10_ IU/mL)	3.78 ± 0.64	3.59 ± 0.49	3.86 ± 0.69	0.310
HBcrAg (log_10_ KU/mL)	5.41 ± 1.23	5.24 ± 1.45	5.48 ± 1.16	0.636
HBV RNA (log_10_ copies/mL)	7.73 ± 1.18	7.72 ± 1.42	7.75 ± 1.09	0.959
HBV cccDNA (copies/cell, median (range))	33.8 (15.4–76.4)	30.0 (13.2–100.8)	34.4 (13.7–66.7)	0.946

### Dynamic changes in HBV RNA, HBV DNA, and cccDNA levels during 48 weeks of peg-IFN treatment

**Fig. 1:**
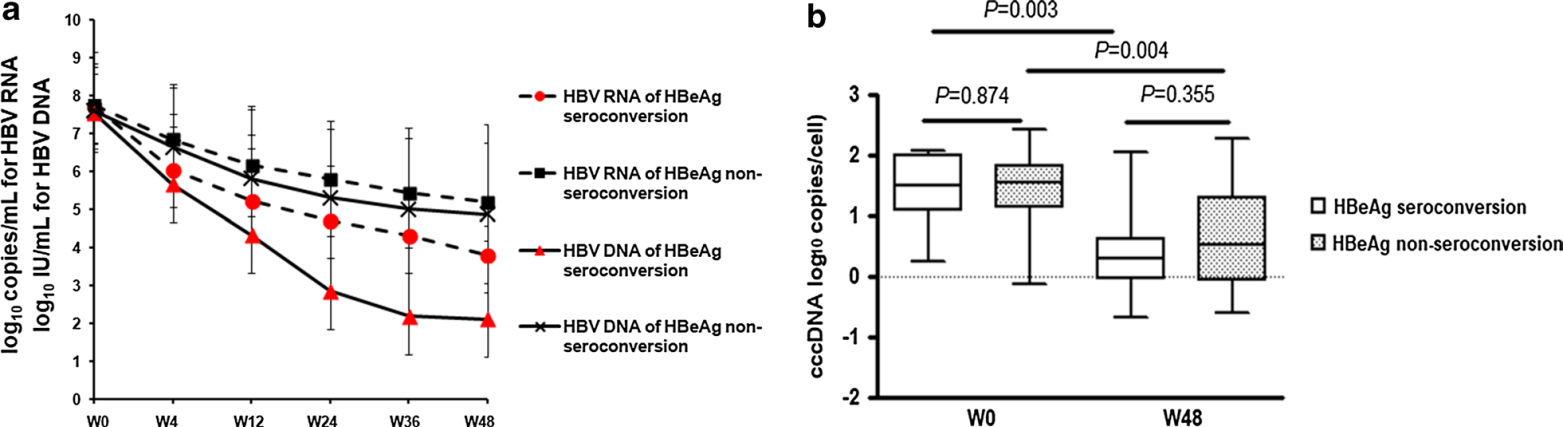
Dynamic changes in serum HBV RNA, HBV DNA (**a**), and cccDNA (**b**) during the 48 weeks of treatment with peg-IFN in the SR group and the NSR group

Among all patients, all the levels of serum HBV RNA, HBV DNA, and intrahepatic cccDNA showed a rapid decline from baseline to week 48 (from 7.73 ± 1.18 log_10_ copies/mL to 4.66 ± 1.56 log_10_ copies/mL for HBV RNA; from 7.57 ± 1.10 log_10_ IU/mL to 4.22 ± 2.45 log_10_ IU/mL for HBV DNA; from median 33.8 copies/cell to 2.53 copies/cell for cccDNA). In SR group, the HBV RNA levels decreased more rapidly than NSR group. At week 48, serum HBV RNA levels were significantly lower in the SR group than the NSR group (3.56 ± 0.64 log_10_ copies/mL vs. 5.00 ± 1.60 log_10_ copies/mL, *P* = 0.002) (Fig. 1a). In the SR group, serum HBV RNA levels decreased more slowly than serum HBV DNA levels, whereas both HBV RNA and HBV DNA levels decreased similarly in the NSR group (Fig. 1a). During the 48-week peg-IFN treatment period, the cccDNA levels decreased significantly from a median value of 30.2 copies/cell to 1.94 copies/cell in the SR group and from a median value of 34.4 copies/cell to 3.23 copies/cell in the NSR group. No difference in cccDNA decline was observed between the SR and NSR groups (Fig. 1b).

### Correlations of cccDNA with HBV RNA, HBcrAg, HBV DNA, and HBsAg before and after 48 -week peg-IFN treatment

**Fig. 2:**
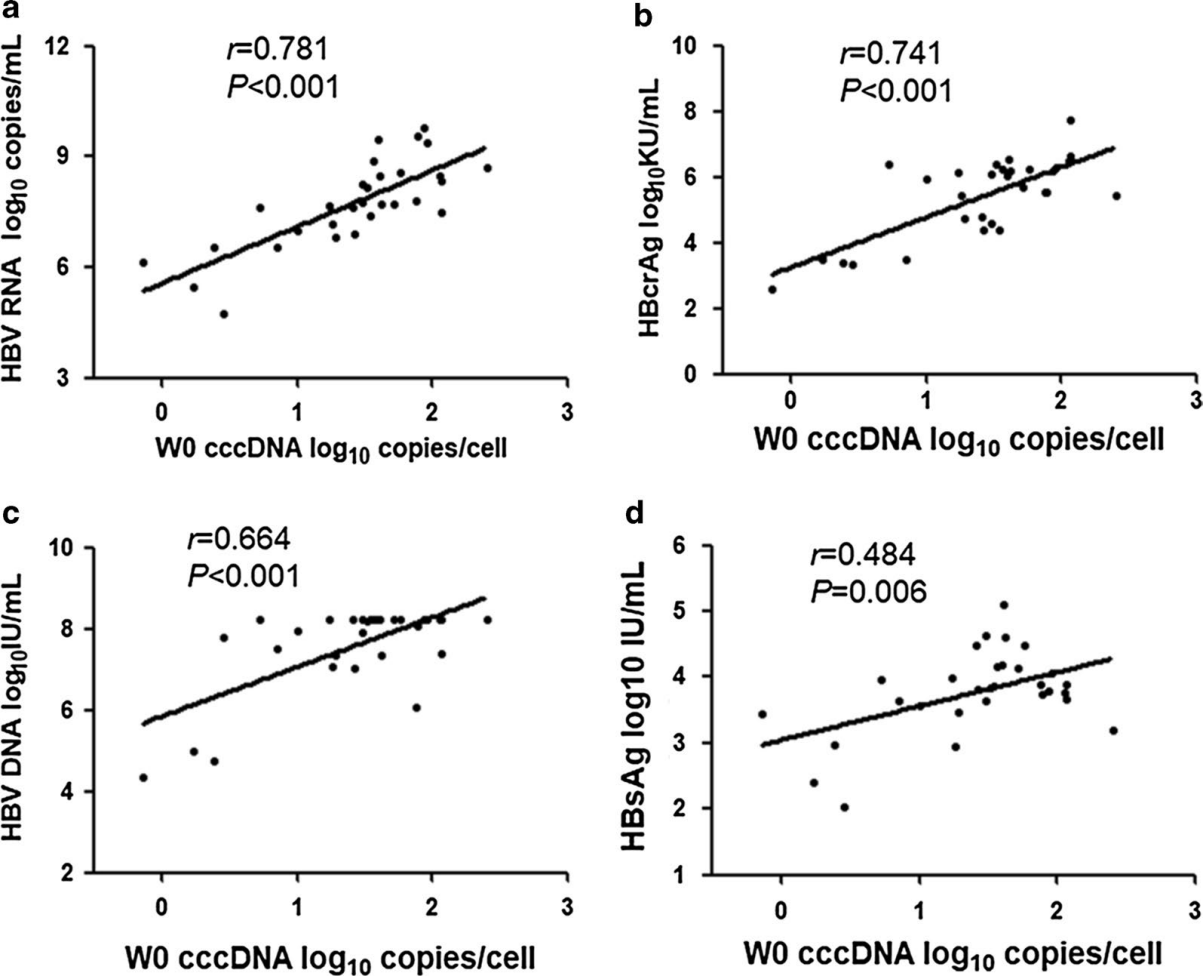
Correlations between the level of intrahepatic cccDNA and the level of serum HBV RNA (**a**), HBcrAg (**b**), HBV DNA (**c**), and HBsAg (**d**) among all patients at baseline. Solid line: linear growth tread; r: correlation coefficient

**Fig. 3:**
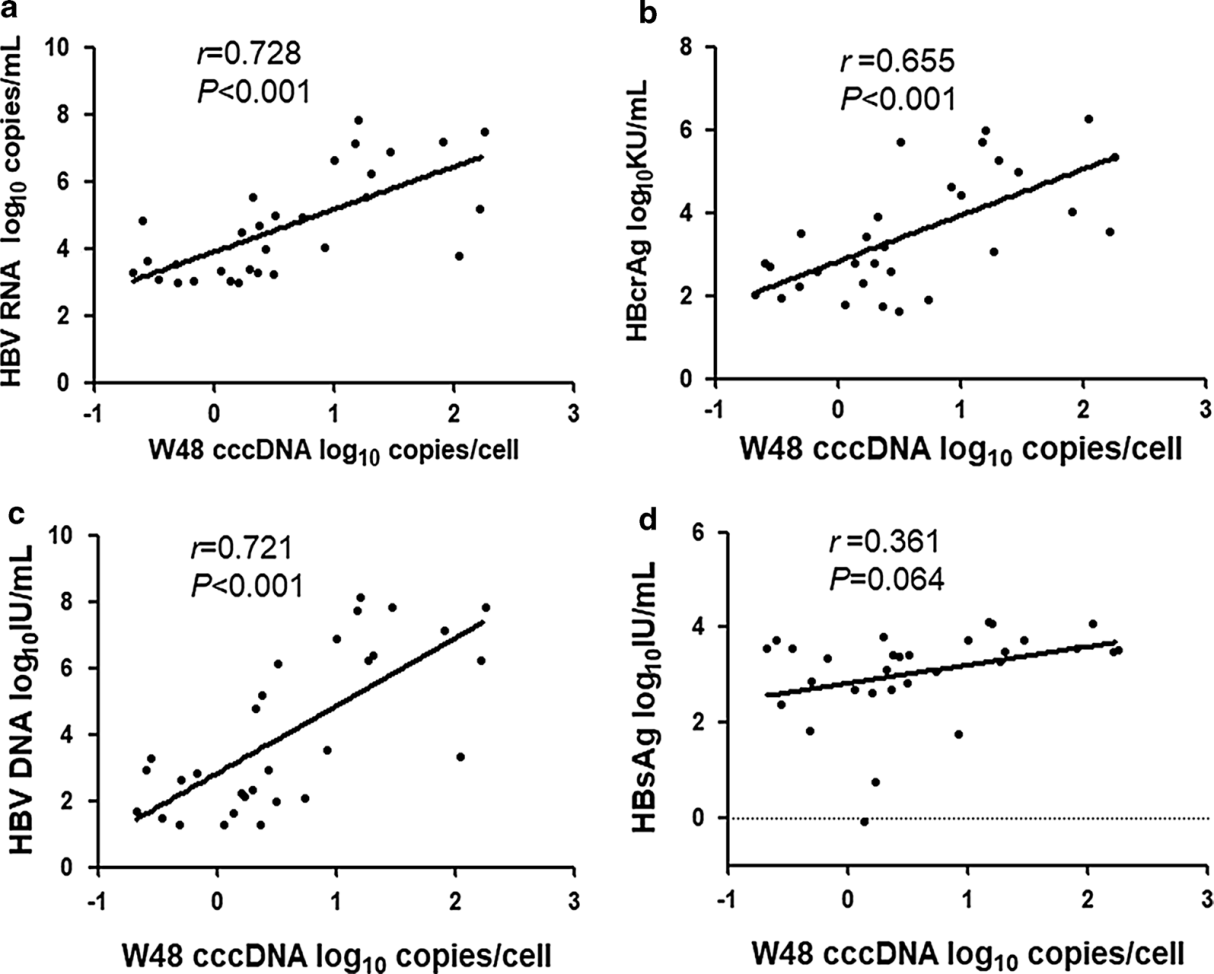
Correlations between the level of intrahepatic cccDNA and the level of serum HBV RNA (**a**), HBcrAg (**b**), HBV DNA (**c**), and HBsAg (**d**) among all patients at week 48. Solid line: linear growth tread; r: correlation coefficient

To further evaluate the performance of HBV RNA, HBcrAg, HBV DNA, HBsAg in reflecting cccDNA profile, the correlation values of each parameter were calculated. Among all patients, intrahepatic cccDNA was positively correlated with serum HBV RNA, HBcrAg, HBV DNA, and HBsAg at baseline. However, cccDNA correlated better with HBV RNA (*r* = 0.781, *P* < 0.001) than with HBcrAg (r = 0.741, *P* < 0.001), HBV DNA (r = 0.664, *P* < 0.001), and HBsAg (*r* = 0.484, *P* = 0.005) (Fig. 2a–d). At 48 weeks after peg-IFN treatment, intrahepatic cccDNA was still significantly correlated with serum HBV RNA (*r* = 0.728, *P* < 0.001, Fig. 3a), HBV DNA(*r* = 0.721, *P* < 0.001, Fig. 3b) and HBcrAg (*r* = 0.655, *P* < 0.001, Fig. 3c), but no correlations were observed between cccDNA and HBsAg levels (*r* = 0.361, *P* = 0.064, Fig. 3d).

**Fig. 4:**
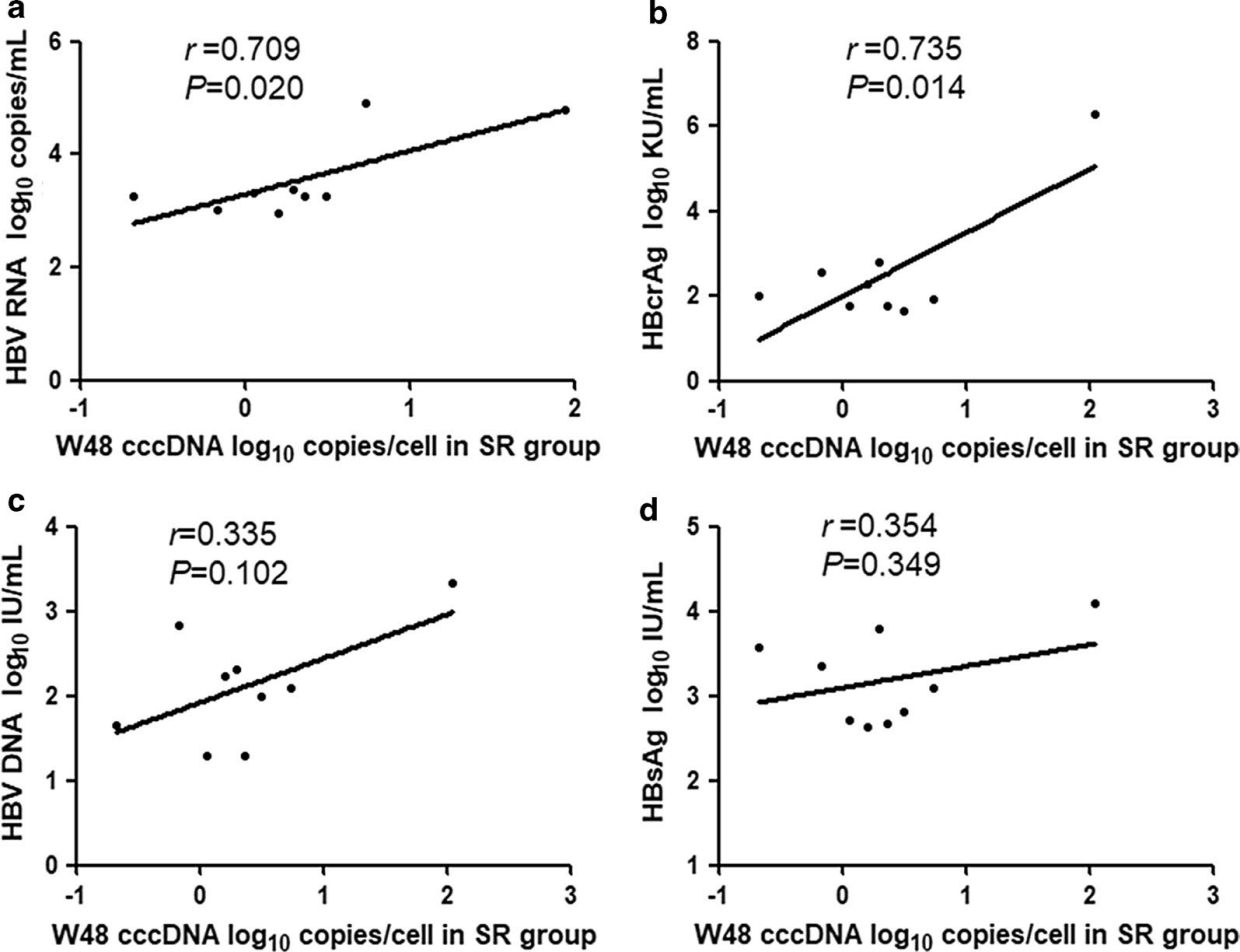
Correlations between the level of intrahepatic cccDNA and the level of serum HBV RNA (**a**), HBcrAg (**b**), HBV DNA (**c**), and HBsAg (**d**) in the SR group at week 48. Solid line: linear growth tread; r: correlation coefficient

**Fig. 5:**
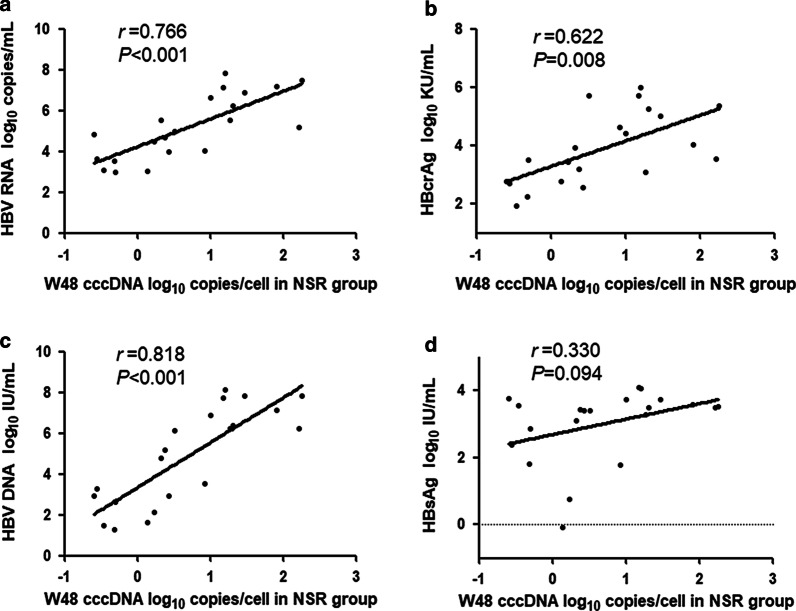
Correlations between the level of intrahepatic cccDNA and the level of serum HBV RNA (**a**), HBcrAg (**b**), HBV DNA (**c**), and HBsAg (**d**) in the NSR group at week 48. Solid line: linear growth tread; r: correlation coefficient

Through analyzing the different groups of CHB patients separately at week 48, we found that in the SR group there were significant positive correlations of intrahepatic cccDNA with serum HBV RNA (*r* = 0.709, *P* = 0.020, Fig. 4a) and HBcrAg (*r* = 0.735, *P* = 0.014, Fig. 4b), but only weak correlations with HBV DNA (*r* = 0.335, *P* = 0.102, Fig. 4c) or HBsAg (*r* = 0.354, *P* = 0.349, Fig. 4d). Different from the SR group, cccDNA correlated strongly with HBV RNA (*r* = 0.766, *P* < 0.001, Fig. 5a) and HBV DNA (*r* = 0.818, *P* < 0.001, Fig. 5c), moderately with HBcrAg (*r* = 0.622, *P* = 0.008; Fig. 5b), but not with HBsAg (*r* = 0.330, *P* = 0.094, Fig. 5d) in the NSR group.

**Fig. 6:**
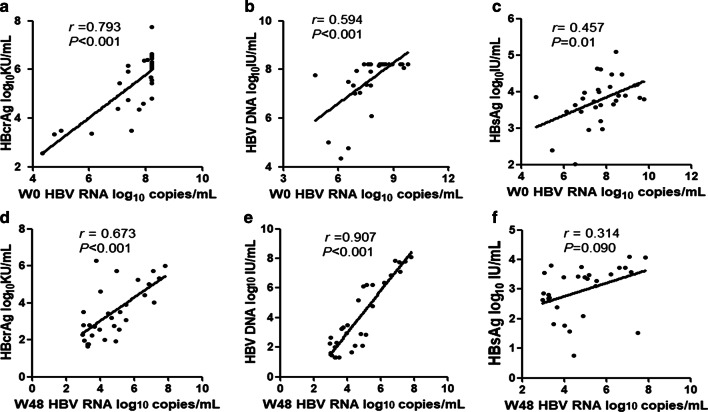
Correlation of serum the level of HBV RNA with HBcrAg (**a**), HBV DNA (**b**), and HBsAg (**c**) among all patients at baseline and with HBcrAg (**d**), HBV DNA (**e**), and HBsAg (**f**) at week 48. Solid line: linear growth tread; r: correlation coefficient

In addition, we also analyzed the correlation between HBV RNA and other serum markers among all patients. The results showed that serum HBV RNA levels strongly correlated with HBcrAg (*r* = 0.793, *P* < 0.001, Fig. 6a) and HBV DNA(*r* = 0.594, *P* < 0.001, Fig. 6b) at baseline, and after 48 weeks peg-IFN treatment the correlations of HBV RNA with HBcrAg (*r* = 0.673, *P* < 0.001, Fig. 6d) and HBV DNA (*r* = 0.907, *P* < 0.001, Fig. 6e) were still positive. However, HBV RNA only moderately correlated with HBsAg both at baseline (*r* = 0.457, *P* = 0.010, Fig. 6c) and after 48 weeks of treatment (*r* = 0.314, *P* = 0.090, Fig. 6f).

## Discussion

In previous studies, serum HBV RNA and HBcrAg have been reported to be potential surrogate biomarkers of cccDNA in predicting the outcomes of peg-IFN or NAs treatments [4, 21, 22]. The correlation of cccDNA with HBV RNA before and after NAs treatment has also been studied [5, 22, 23]. However, it is still unclear whether serum HBV RNA or HBcrAg is a better marker of cccDNA in patients treated with peg-IFN.

With the aim to determine the best surrogate marker of intrahepatic cccDNA during peg-IFN therapy, we analyzed the correlations of intrahepatic cccDNA with serum HBV RNA, HBcrAg, HBV DNA, and HBsAg before and after peg-IFN treatment in HBeAg-positive CHB patients. Additionally, the kinetics of HBV RNA and intrahepatic cccDNA among all patients were analyzed.

The results showed that there was no difference of serum HBV RNA or HBcrAg in patients with and without HBeAg seroconversion at baseline (all *P* > 0.05), which indicated that HBV RNA and HBcrAg at baseline may be not associated with HBeAg-seroconversion in HBeAg-positive patients treated with peg-IFN. During peg-IFN treatment, HBV RNA declined more rapidly in patients with HBeAg seroconversion than those without HBeAg seroconversion. After 48-week peg-IFN treatment, serum HBV RNA declined smaller than serum HBV DNA in the SR group, whereas both of them decreased parallel in the NSR group. Our study showed that peg-IFN could profoundly reduce the load of cccDNA after 1 year of treatment, which was similar to previous studies [24, 25]. Besides, we found that the cccDNA decreased more in the SR group than that in the NSR group. We deduced that the discrepancy might be as a result of peg-IFN suppressing the replication and transcription of cccDNA, which resulted in the increasing clearance rates of HBV RNA, HBcrAg, and cccDNA [26].

Our study also showed that intrahepatic cccDNA levels correlated best with serum HBV RNA (*r* = 0.781, *P* < 0.001) than with HBcrAg (*r* = 0.741, P < 0.001), HBV DNA (*r* = 0.664, *P* < 0.001), and HBsAg (*r* = 0.484, *P* = 0.006) at baseline. After 48 weeks of peg-IFN treatment the correlation between cccDNA and HBV RNA was still strong (*r* = 0.728, *P* < 0.001), while HBsAg did not correlate with cccDNA (*r* = 0.361, *P* = 0.064). These data indicated that serum HBV RNA might be a superior serological marker of intrahepatic cccDNA during peg-IFN treatment. In Gao’s previous study, serum HBV RNA was identified only having a weak correlation with cccDNA in a 96-week NAs treatment, which was different from our results [5]. The difference may be attributed to the different antiviral mechanisms of NAs and peg-IFN [27]. Besides, an animal experiment in HBV-infected mice indicated that there was no correlation between serum HBV RNA and intrahepatic cccDNA after 6 weeks of peg-IFN therapy [12]. The discrepancies might because the mouse model could not fully reflect the real conditions in clinical patients.

A few reports believed that serum HBcrAg was better than HBV RNA in reflecting intrahepatic cccDNA levels in treatment-naive patients [17, 28]. However, our study showed that the correlation of cccDNA with HBcrAg became weaker than with HBV RNA after 48 weeks of peg-IFN therapy, although HBcrAg and HBV RNA have a similar correlation with cccDNA at baseline.

We further compared the correlations of cccDNA with HBV RNA, HBV DNA, and HBsAg between patients with and without HBeAg seroconversion after 48 weeks of peg-IFN treatment. The results showed that HBV RNA and HBcrAg were positively correlated with cccDNA both in the SR and NSR groups at week 48 (HBV RNA: *r* = 0.709, *P* = 0.002 in the SR group; *r* = 0.766, *P* < 0.001 in the NSR group. HBcrAg: *r* = 0.735, *P* = 0.014 in the SR group; *r* = 0.662, *P* = 0.008 in the NSR group). HBV DNA was strongly correlated with cccDNA in the NSR group (*r* = 0.818, *P* < 0.001), but no positive correlation was observed in the SR group (*r* = 0.335, *P* = 0.102). The correlation between HBsAg and cccDNA was not found in either the SR group (*r* = 0.354, *P* = 0.349) or the NSR group (*r* = 0.374, *P* = 0.094). The results indicated that HBV RNA and HBcrAg were better markers than HBV DNA and HBsAg in reflecting cccDNA no matter the results of antiviral treatment.

Although previous studies have shown that serum HBsAg quantification may reflect the level of intrahepatic cccDNA both in HBeAg-positive and HBeAg-negative CHB patients [29, 30], our study showed that cccDNA weakly correlated with HBsAg before treatment and did not correlate with HBsAg after 48 weeks of peg-IFN therapy. This pattern possibly because HBsAg could be produced not only from cccDNA in infected hepatocytes but also from viral sequences integrated into the host genome [31]. These data indicated that serum HBsAg level could not accurately reflect the level of intrahepatic cccDNA in the cohort of our study.

Our results also suggested that serum HBV RNA correlated well with HBcrAg at baseline and at week 48 with peg-IFN treatment. As we know, HBcrAg was translated from preC mRNA which was also transcribed from cccDNA, and thus HBcrAg may have a better correlation intensity with HBV RNA [17]. HBV RNA showed a better correlation with serum HBV DNA at week 48 than at baseline (*r* = 0.907 vs. *r* = 0.594). For those HBV DNA with baseline levels higher than the upper detection range 1 × 10^8^ IU/mL (8log10 IU/mL), their values were defined as 8log 10 IU/mL. The data adjustment may slightly affect the correlation of HBV DNA and HBV RNA at baseline. Our results also showed that the HBV RNA level decreased significantly, whereas the HBsAg level remained stable after 48 weeks of treatment. As a result, the correlation between HBV RNA and HBsAg (*r* = 0.314, *P* = 0.090) after 48 weeks of peg-IFN treatment was not significant although they had a moderate correlation at baseline (*r* = 0.457, *P* = 0.01).

Several limitations exist in this study. Due to the difficulty of acquiring liver samples both at baseline and after 48 weeks of peg-IFN therapy, the sample size in our study was relative small and the patients enrolled were mainly those infected with HBV genotype B or C. So studies on larger sample size cohort are required to confirm the present findings in the future.

## Conclusions

Both Serum HBV RNA and HBcrAg correlated significantly with intrahepatic cccDNA before and after 48-week peg-IFN treatment in HBeAg-positive patients. However, serum HBV RNA correlated with cccDNA more strongly than HBcrAg, HBV DNA, and HBsAg, irrespective of treatment results. HBV RNA also correlated well with HBcrAg before and after peg-IFN therapy. The study indicated that serum HBV RNA might be an ideal surrogate non-invasive marker in reflecting the intrahepatic cccDNA profile during peg-IFN treatment.

## Data Availability

The data analyzed in the current study is available from the corresponding author on reasonable request.

## Abbreviations

HBV: Hepatitis B virus
HBV RNA: Hepatitis B virus RNA
HBcrAg: HBV core-related antigen
ALT: Alanine aminotransferase
cccDNA: Covalently closed circular DNA
CHB: Chronic hepatitis B
HBeAg: Hepatitis B e antigen
HBsAg: Hepatitis B surface antigen
Peg-IFN: Pegylated interferon
NAs: Nucleotide/nucleoside analogs
